# Length-Dependent Prolongation of Force Relaxation Is Unaltered by Delay of Intracellular Calcium Decline in Early-Stage Rabbit Right Ventricular Hypertrophy

**DOI:** 10.3389/fphys.2017.00945

**Published:** 2017-12-04

**Authors:** Michelle M. Monasky, Carlos A. A. Torres, Paul M. L. Janssen

**Affiliations:** ^1^Department of Physiology and Cell Biology, College of Medicine, Ohio State University, Columbus, OH, United States; ^2^Department of Emergency Medicine, Ohio State University, Columbus, OH, United States

**Keywords:** preload, myofilament, calcium handling, hypertrophy, rabbit

## Abstract

Chronic pressure overload can result in ventricular hypertrophy and eventually diastolic dysfunction. In normal myocardium, the time from peak tension to 50% relaxation of isolated cardiac myocardium is not directly determined by the time for calcium decline. This study aims to determine whether the time for calcium decline is altered with a change in preload in early-stage hypertrophied myocardium, and whether this change in time for calcium decline alters the rate of relaxation of the myocardium. Young New Zealand white rabbits underwent a pulmonary artery banding procedure and were euthanized 10 weeks later. Twitch contractions and calibrated bis-fura-2 calcium transients were measured in isolated thin right ventricular trabeculae at optimal length and with the muscle taut. Systolic calcium, calcium transient amplitude, and time from peak tension to 50% relaxation all increased with an increase in preload for both hypertrophied and sham groups. Time for intracellular calcium decline increased both with an increase in preload and an increase in extracellular calcium concentration in hypertrophied myocardium but not in sham, while time from peak tension to 50% relaxation did not significantly change between groups under either condition. Also, time for intracellular calcium decline generally decreased with an increase in extracellular calcium for both hypertrophied and sham groups, while time from peak tension to 50% relaxation generally did not significantly change in either group. Combined, these results indicate that the mild hypertrophy significantly changes calcium handling, but does not impact on the rate of force relaxation. This implies that the rate-limiting step in force relaxation is not directly related to calcium transient decline.

## Introduction

Chronic pressure overload can result in ventricular hypertrophy and eventually diastolic dysfunction. While initially the hypertrophic response is compensatory, it can progress to a decompensatory response (Faber et al., 2006) and eventually lead to heart failure (Pokharel et al., 2003). Attempts to reduce ventricular load through use of diuretics and vasodilators have not been fully adequate in reducing left ventricular mass (Pokharel et al., 2003). The right ventricle is also affected by hypertrophy, as it is subjected to abnormal loading conditions in many patients with congenital heart disease (Faber et al., 2006). Pulmonary artery banding in rabbits has been shown to also induce a degree of left ventricular diastolic dysfunction (Kitahori et al., 2009). Diastolic dysfunction can impair ventricular filling, and can be a major contributor to the cardiac dysfunction in heart failure (Grossman, 1990). In a model of rabbit right ventricular hypertrophy we have shown that diastolic dysfunction was observed 10 weeks after banding (Varian et al., 2009). Since calcium handling is known to be altered during hypertrophy, it is currently unclear whether, and to what extent, under near physiological conditions the intracellular calcium transient amplitude and kinetics contribute to the length-dependent increase in force and increase in duration of relaxation under early pathological conditions.

The rate of myocardial relaxation in normal myocardium has recently been shown to be complex, and involves a large number of molecular pathways, proteins, and post-translational modifications, for a recent review see Biesiadecki et al. (2014). Relaxation prominently involves myofilament properties, rather than solely reflect on the rate of calcium decline (Monasky et al., 2008). The heart's ability to increase cardiac output as a result of an increase in venous return is known as the Frank-Starling mechanism (Shiels and White, 2008; de Tombe et al., 2010). This increase in ventricular end-diastolic pressure and volume increases the amount of stretching the chamber has to do to accommodate the increase in blood volume. Preload thus plays a major role in the modulation of contraction and relaxation of myocardium. Within the *in vivo* pre-load range, as muscle length is increased, relaxation will become prolonged (Janssen and Hunter, 1995) and more force will be produced.

In general, we aimed this study at determining whether rate-limiting steps in force relaxation are impacted in early-stage hypertrophy. This study specifically aims to determine whether the time for calcium decline is altered with a change in preload in the pulmonary artery banding animals when compared to sham-operated animals, and whether a change in time for calcium decline will alter the force-relaxation time of the myocardium, in order to further understand the rate-limiting processes of cardiac relaxation, as they play a large role in cardiac dysfunction. We found that while the time for 50% calcium decline is longer in the banded animals compared to the sham operated animals, the time from peak tension to 50% relaxation is not significantly different between banded and sham-operated animals.

## Materials and methods

This study was approved by the IACUC of the Ohio State University. All animal procedures were conducted in accordance with guidelines published in *the Guide for the Care and Use of Laboratory Animals*.

### Pulmonary artery banding procedure

Young male New Zealand white rabbits (*n* = 16, 1–1.5 kg), were sedated with acepromazine (1.25 mg/kg, subcutaneously) and anesthetized with isoflurane (5% in anesthetic chamber). Animals received 100% oxygen (at a rate of 400–600 mL/min) and isoflurane (at a rate of 0.5–1%) through a loose-fitting face mask designed for a small animal. Chloramphenical (30 mg/kg) was administered subcutaneously prior to surgery. Rabbits were placed in dorsal recumbency, and surgical anesthesia was confirmed by the absence of the pedal reflex. Standard limb lead ECGs and pulse oximeter were obtained during the period of surgery. The skin over the sternum was denuded of hair and prepared aseptically. The incision line was infiltrated with 2% lidocaine. A midline incision (~3–4 cm) was made in the skin, and the sternum was split with a #10 scalpel blade, being careful to remain precisely on the midline so that the pericardial sac could be observed, and the sac was incised to reveal the surface of the pulmonary artery. The pulmonary was ligated to a piece of polyethylene tube (O.D. 3.20 mm) and the tube was removed. Ligature was performed using 4/0 monofilament polypropylene suture. The pericardial sac was left open, but the sternum was closed with three simple interrupted sutures of 3/0 monofilament polydioxanone suture. Muscle layers were closed with simple interrupted suture of 4/0 nylon, and the subcutaneous was closed with simple interrupted suture of 4/0 polyglactin. All rabbits were given 0.01 mg/kg buprenorphine IM, once after the end of surgery and 30 mg/kg chloramphenicol SC, BID for 3 days postoperatively. Sham-operated animals were treated and handled identically, with the sole omission of placing the band around the pulmonary artery. All of the animals survived the surgery and recovery. The degree of hypertrophy was assessed using the weight of the right ventricle after 10 weeks as banding as a primary indicator of hypertrophy.

### Measurement of twitch contractions and calcium transients

At 10 weeks post banding, rabbits were injected with 5,000 units/kg Heparin and intravenously anesthetized with 50 mg/kg pentobarbital sodium. The chest was opened by bilateral thoracotomy, the heart rapidly excised, and ultra-thin trabeculae (average dimensions 234 ± 23 μm wide, 149 ± 15 μm thick, and 3.05 ± 0.29 mm long) dissected from the right ventricle. The muscles were dissected in a Krebs-Henseleit solution containing (in mM) 137 NaCl, 5 KCl, 1.2 MgSO_4_, 1.2 NaH_2_PO_4_, 20 NaHCO_3_, 10 glucose, and 0.25 CaCl_2_. 20 mM of 2,3-butanedione monoxime (BDM) was added to this solution to minimize cutting damage and to arrest the heart (Mulieri et al., 1989). Muscles were mounted in the setup as previously described (Monasky et al., 2008; Billman et al., 2010) and stimulated at 2 Hz while perfused with an oxygenated Krebs-Henseleit solution, now without BDM and containing 1 mM Ca^2+^. The muscles were stretched until an increase in passive (diastolic) force was no longer accompanied by a substantial increase in developed force. The muscles were allowed time to equilibrate (~20 min, or until twitches were consistent). Previous studies have shown this length (i.e., optimal length) to correspond to a sarcomere length of about 2.2 μm, which approximates the end-diastolic sarcomere length in the *in vivo* beating heart (Rodriguez et al., 1992).

Trabeculae (*n* = 5 banded, *n* = 6 sham, *n* = 1 per rabbit) were iontophoretically loaded with the fluorescent calcium indicator bis-fura-2 as previously described (Backx and Ter Keurs, 1993; Layland and Kentish, 1999; Hiranandani et al., 2006; Monasky et al., 2008). Briefly, after assessment of back-ground fluorescence, a micro-pipette is loaded with bis-fura-2, and gently impaled into a single cell of the multicellular preparation. A negative current is applied to the pipette, slowly drawing the bis-fura-2 into this cell, from which the dye slowly spreads, via gap junctions, to neighboring myocytes. After ~30 min of loading (the muscle is not contracting at this time, but oxygenated solution is circulating), the pipette is removed (sometime a second dye-loading site is loaded if the muscle is particularly long), and stimulation is reinstated. After 20 min, the dye has uniformly spread into the myocytes of this multicellular trabecular. At the conclusion of the experiment, the muscle is semi-permeabilized, and solutions with a known calcium concentration are used to calibrate the muscle. At the end of the calibration procedure, all dye is quenched, and background fluorescence is assessed. From the other rabbits (*n* = 5), either no suitable trabeculae was found, or the entire experimental protocol could not be completed. Force-development and simultaneously obtained calcium transients were measured at various extracellular calcium concentrations at taut (i.e., not stretched yet not slack) and optimal (stretched until an increase in developed force is accompanied by a disproportional increase in diastolic force) muscle lengths. At 1.0 mM [Ca^2+^]_o_, force and calcium transient amplitude were measured at each of the lengths after forces had stabilized at each length. The muscle was then returned to the taut position using the micrometer reading as a guide to assure accuracy in returning to the same length, and [Ca^2+^]_o_ was increased to 2.5 mM. Time was allowed for the new calcium to circulate throughout the setup, and force and calcium transients were again measured at each of the prior lengths. The same procedure was repeated at 4.0 mM [Ca^2+^]_o_.

Since bis-fura-2 to some extent leaked and/or was photobleached during the experiments, especially at body temperature, it was not possible to obtain an *in vivo* K_d_, because this procedure would extend the experimental time by an hour, within which the dye concentration would have fallen too much to obtain unambiguous calibrations. However, in order to calibrate each experiment, we obtained an R_min_ and R_max_ containing a calcium chelating agent (EGTA) and high calcium, respectively, and used those values together with the published K_d_-value for bis-fura-2 (370 nM) to construct a curve of Ca^2+^ concentration vs. the ratio of 340/380. By using the equation [Ca^2+^]_i_ = K′[R–R_min_]/[R_max_-R] where K′ = 380(no Ca^2+^)/380(max Ca^2+^)^*^K_d_, we calculated the amount of [Ca^2+^]_i_ that corresponds with our fluorescent ratios (Backx and Ter Keurs, 1993).

Twitch contractions were continuously recorded throughout the experiment. Force development was normalized to the cross sectional area of the trabeculae to allow for comparison between muscles of different diameters. Twitches were recorded at each experimental condition upon stabilization of developed tension. Data were collected and analyzed using in-house software (LabView, National Instruments). The overall protocol was kept as short as possible, to avoid complications of unavoidable, time-dependent drift of intracellular calcium transients (Milani-Nejad et al., 2014).

### Statistics

Data were statistically analyzed using repeated measures ANOVA followed by Tukey's HSD test where applicable. Single data points, such as heart weight, were assessed by simple unpaired *t*-test. A two-tailed value of *P* < 0.05 was considered significant. Data are represented as mean ± SEM.

## Results

In the banded rabbits, the right ventricular weight was increased compared to both body weight (in g/kg) and total heart weight (in g/g) in sham-operated rabbits (0.72 ± 0.02 vs. 0.52 ± 0.03, *P* < 0.05, and 0.24 ± 0.02 vs. 0.19 ± 0.01, *P* < 0.05, *t*-test). The slightly younger age of the rabbits used in this study, combined with the use of the size of the band, caused this degree of hypertrophy to be slightly less than in our previous experiments, in which RV/BW and RV/HW was 0.84 ± 0.04 and 0.27 ± 0.02, respectively (Varian et al., 2009). Moreover, the right atrial weight in these animals was no longer significantly increased vs. sham animals (0.13 ± 0.01 vs. 0.11 ± 0.01, RA/BW, in g/kg), while this was previously significantly larger (0.16 ± 0.01, *P* < 0.05, *t*-test). This indicates there is a significant, but mild hypertrophy only in these banded rabbits.

**Figure 1:**
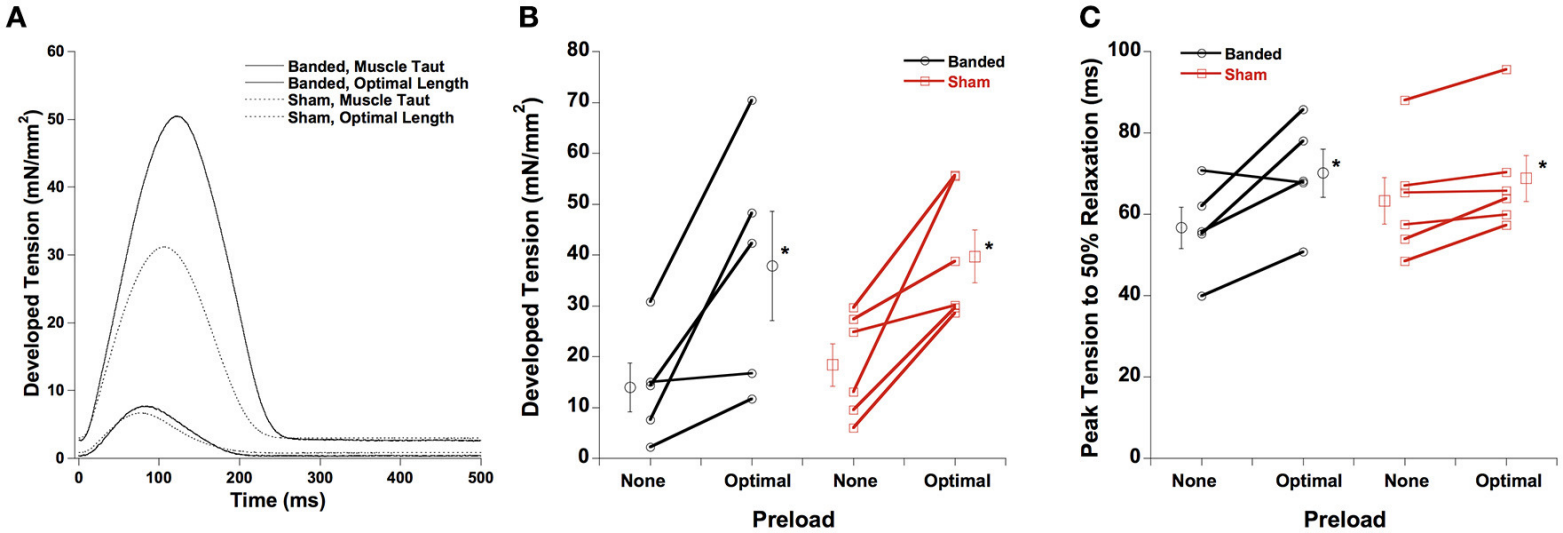
Representative force tracings at taut and optimal muscle length **(A)**. Developed force **(B)** and peak tension to 50% relaxation **(C)** are increased at a higher preload in both sham and banded animals, and are not significantly different between surgical groups. Data collected at 2.5 mM [Ca^2+^]_o_, 37°C. ^*^P < 0.05 vs. no preload of the same group.

A representative tracing for developed force and time from peak tension to 50% relaxation is shown in Figure 1A, with averaged data in Figures 1B,C, respectively. Both developed force and peak tension to 50% relaxation are increased at a longer muscle length (optimal preload) compared to the shorter muscle length (no preload) in both sham and banded animals, and are not significantly different between surgical groups at either of the preloads investigated. Similarly, not shown, time to peak tension is prolonged in both groups at higher length, but again no differences are found between surgical groups.

**Figure 2:**
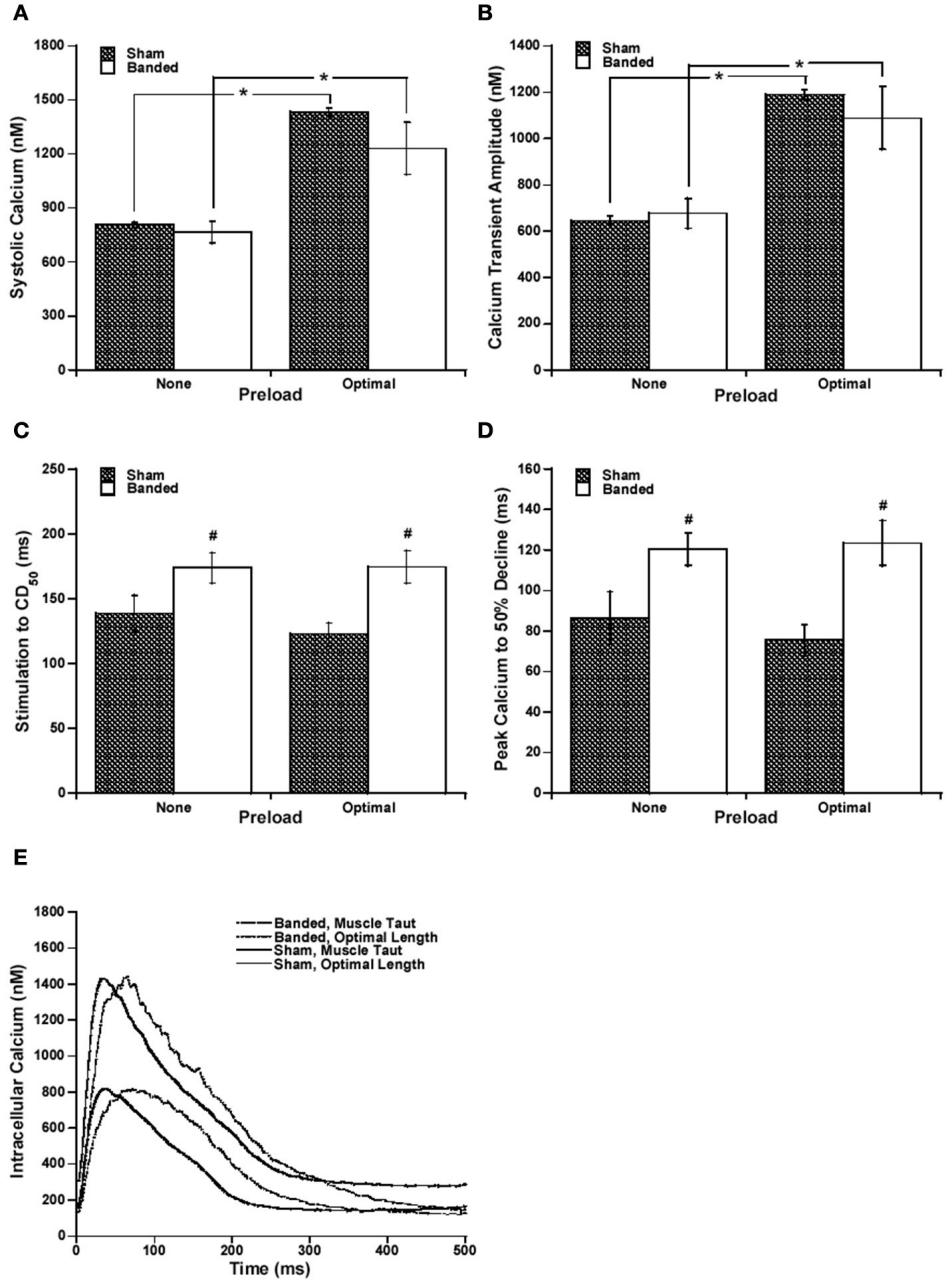
Representative intracellular calcium tracings at taut and optimal muscle length **(A)**. Systolic calcium **(B)** and calcium transient amplitude **(C)** are increased at a higher preload in both sham and banded animals, and are not significantly different between surgical groups. Stimulation to CD_50_ **(D)** and peak calcium to 50% decline **(E)** do not significantly change with a change in preload, but are both significantly increased in banded animals compared to sham at both preloads studied. Data collected at 2.5 mM [Ca^2+^]_o_, 37°C. Sham-operated representative tracing experiment number 090706, banded representative tracing experiment number 090805. ^*^P < 0.05 vs. no preload, ^#^*P* < 0.05 vs. SHAM.

Because it has been previously shown that calcium handling is often altered in hypertrophy and heart failure (Gwathmey and Morgan, 1985; Gwathmey et al., 1987; Yano et al., 2005), we measured intracellular calcium. Systolic calcium and calcium transient amplitude, as seen in Figures 2A,B, respectively, are increased at a higher preload compared to the lower preload in both sham and banded animals. However, both the systolic calcium level and cytosolic calcium transient amplitude are not significantly different between surgical groups at either of the preloads measured.

**Figure 3:**
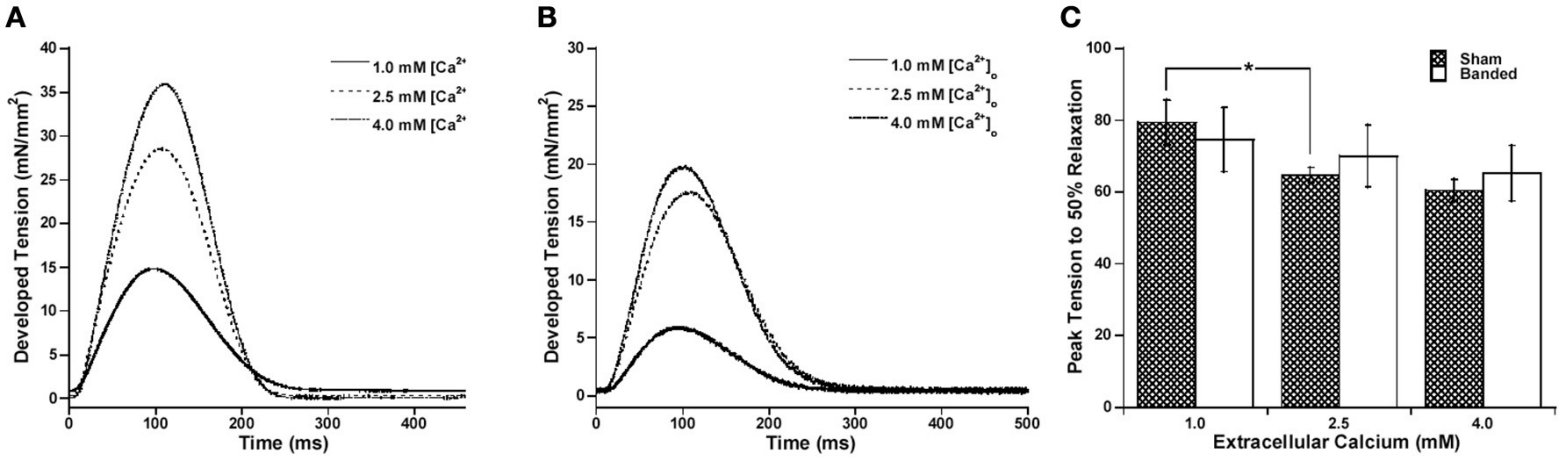
Representative force tracings at 1.0, 2.5, and 4.0 mM [Ca^2+^]_o_ for sham **(A)** and banded **(B)** animals. Peak tension to 50% relaxation significantly decreases in sham from 1.0 to 2.5 but does not significantly change from 2.5 to 4.0 mM extracellular calcium, and does not significantly change at any of the extracellular calcium concentrations measured in the banded animals **(C)**. Data collected at 2.5 mM [Ca^2+^]_o_, 37°C. Sham-operated representative tracing experiment number 081114, banded representative tracing experiment number 090312. ^*^P < 0.05.

**Figure 4:**
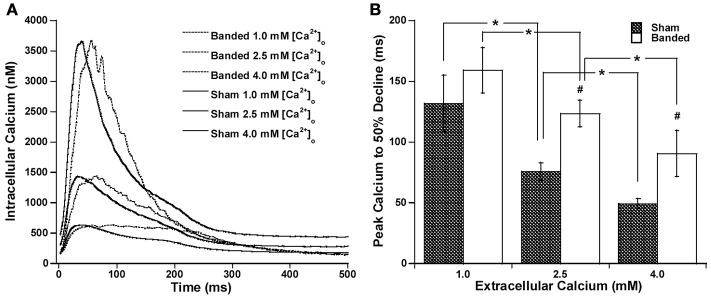
Representative calcium transient tracings at optimal length in banded and sham-operated animals at 1.0 mM, 2.5, and 4.0 mM extracellular calcium concentration **(A)**. Peak calcium to 50% decline significantly decreases from 1.0 to 2.5 mM and from 2.5 to 4.0 mM in both banded and sham **(B)**. Also, peak calcium to 50% decline was significantly greater in banded compared to sham at 2.5 and 4.0 mM extracellular calcium concentration. Data collected at 2.5 mM [Ca^2+^]_o_, 37°C. Sham-operated representative tracing experiment number 090706, banded representative tracing experiment number 090805. ^*^P < 0.05, ^#^*P* < 0.05 vs. SHAM.

Since the calcium transient is often prolonged in hypertrophy and heart failure (Milani-Nejad and Janssen, 2014), and also it has been shown that in normal myocardium force decline is not altered by calcium decline, we investigated whether the time for calcium decline was altered in the pulmonary artery banding model, and whether this would affect the time for force decline. Figure 2 demonstrates that stimulation to 50% of calcium decline (Figure 2C) and peak calcium to 50% decline (Figure 2D) do not significantly change with a change in preload, but are both significantly increased in banded animals compared to sham at both preloads studied. Figure 2E shows representative calcium tracings. Figure 3 shows that the time from peak tension to 50% relaxation significantly decreases in the sham animals when the extracellular calcium is increased from 1.0 to 2.5 mM but does not significantly change when the extracellular calcium is increased from 2.5 to 4.0 mM. Additionally, the time from peak tension to 50% relaxation is not significantly different between banded and sham animals at any of the extracellular calcium concentrations measured (Figure 3C). However, the time from peak calcium to 50% decline significantly decreases in both banded and sham when the extracellular calcium is increased from 1.0 to 2.5 mM and from 2.5 to 4.0 mM (Figure 4B). Also, the time from peak calcium to 50% decline was significantly longer in banded compared to sham animals at 2.5 and 4.0 mM extracellular calcium concentration. Thus, the time from peak tension to 50% relaxation was not significantly altered despite a significantly prolonged calcium transient in the banded animals compared to the sham animals. Representative calcium transient tracings at optimal length in banded and sham-operated animals at 1.0, 2.5, and 4.0 mM extracellular calcium concentrations are shown in Figure 4A.

## Discussion

The major finding in this study is that time for force relaxation is not significantly different between banded and sham-operated animals, despite a longer time for calcium transient decline in the early-stage right ventricular hypertrophied myocardium. This finding is in agreement with a previous study which found length-dependent prolongation of relaxation at longer muscle lengths compared to shorter muscle lengths, despite no significant change in the time for calcium decline between the different muscle lengths (Monasky et al., 2008). These results confirm that the myofilaments play a prominent role prominent role in the rate of relaxation in normal, large mammalian myocardium (Biesiadecki et al., 2014), and add to this that they remain prominent determinants of relaxation in mildly hypertrophied myocardium where calcium handling changes have already occurred.

The pulmonary artery banding model is well established, and has been used in dogs for 8 months (Visner et al., 1986), cats for 11 weeks(Quaile et al., 2007), young lambs for 8 weeks (Leeuwenburgh et al., 2008), rabbits for 4 and 10 weeks (Varian et al., 2009), and rats for 3 weeks (Braun et al., 2003). In our rabbit model, after 10 weeks of banding the animals did not exhibit clinical signs of heart failure, such as peripheral edema or lethargy, and are not yet in the failing stage. Still, the right ventricle had already significantly hypertrophied while the left ventricle is unaffected, as previously shown (Varian et al., 2009). In this early-stage hypertrophied myocardium, the main findings in this study include that time from peak calcium to 50% calcium decline was significantly prolonged compared to sham-operated animals at both a low and high preload, and when the extracellular calcium concentration was 2.5 and 4.0 mM. However, the time from peak tension to 50% force-relaxation was not significantly different between banded and sham animals at either preload or at any extracellular calcium concentration investigated. Previously, we showed that when this hypertrophy is larger in this model (Varian et al., 2009), force-relaxation can become significantly affected. This study on only mildly right-ventricular hypertrophic rabbits allowed us to show calcium handling changes have occurred without yet a significant impact on the rate of force decline. This suggests that the myofilaments predominantly determine the rate of relaxation, even in the presence of a delayed intracellular calcium decline.

We chose to use right ventricular trabeculae for this study for a number of reasons. First, the right ventricle is subjected to abnormal loading conditions in many patients with congenital heart disease (Faber et al., 2006). In right ventricular hypertrophy, the myocardium experiences a significant increase in wall thickness, a negative force-frequency, an increase in diastolic tension, downregulation of SERCA, upregulation of NCX, and upregulation of ANP (Varian et al., 2009), similar to changes that occur in left ventricular hypertrophy (Hasenfuss et al., 1994, 1996, 1999; Prestle et al., 1999). Therefore, the results obtained from the right ventricular trabeculae will likely resemble to large degree the results that would have been obtained from a similar study in the left ventricle. Second, linear trabeculae of a certain size are required as an experimental limitation (Schouten and ter Keurs, 1986; Raman et al., 2006), and these are typically found in much greater abundance in the right ventricle as opposed to the left. Therefore, by using right ventricular trabeculare, many fewer rabbits were needed, and this greatly increased the feasibility of the project. The systemic impact of right ventricular hypertrophy is less than a similar degree of banding on the left ventricle. This thus provides a model in which systemic changes that feed-back on the contractile properties are less of a confounding factor, and the results are more likely to be a cause of the hypertrophy then on the systemic reaction to a loss of cardiac output.

The slower force kinetics, both time to peak tension and 50% relaxation time are prolonged in muscles from both sham and banded animals. The slower force decline is likely due to a direct impact on cross-bridge kinetics. With increased length of the muscle, reflecting increase ventricular preload, the cross-bridge attachment rate is slowed (Milani-Nejad et al., 2013, 2015). In addition, the higher ATP demand upon increased preload may increase ADP, and/or decrease the ATP/ADP ratio, which slows down cross-bridge detachment (Schoenberg and Eisenberg, 1987). On the molecular level, it has recently been shown that phosphorylation of tropomyosin, troponin I, and myosin light chain-2 is increased at a high preload compared to no preload (Monasky et al., 2010, 2013). Another study showed that frequency-dependent myofilament desensitization does not occur in this model and that this may be partially due to the loss of troponin I frequency-dependent phosphorylation (Varian et al., 2009). Future studies are needed to investigate whether length-dependent myofilament protein phosphorylation is potentially altered in this model. However, this is considered beyond the scope of the current study, since in order to determine phosphorylation status differences, the trabeculae need to be flash frozen while twitching for later molecular analysis. This was not possible with the trabeculae in this study because the calcium-binding fluorescent indicator calibration protocol requires that the muscle membrane be permeabilized at the end of the experiment, which renders the muscle useless for unambiguous phosphorylation studies.

In conclusion, length-dependent prolongation of force relaxation is unaltered by a delay in the intracellular calcium decline in this early-stage rabbit right ventricular hypertrophy model. Thus, the myofilaments are predominantly responsible for the rate of relaxation in both normal and early-stage hypertrophied myocardium, and should be further investigated as potential therapeutic targets.

## Author contributions

MM and CT: performed experiments and data analysis; MM: drafted manuscript; MM, CT, and PJ: edited manuscript; PJ designed experiments; MM, CT, and PJ interpreted data.

### Conflict of interest statement

The authors declare that the research was conducted in the absence of any commercial or financial relationships that could be construed as a potential conflict of interest.
